# Childhood Anxiety Symptoms as a Predictor of Psychotic Experiences in Adolescence in a High-Risk Cohort for Psychiatric Disorders

**DOI:** 10.1093/schizbullopen/sgae003

**Published:** 2024-04-15

**Authors:** Viviane Machado, Lais Fonseca, Matheus Ghossain Barbosa, Rodrigo A Bressan, Pedro Pan, Luis Augusto Rohde, Euripedes Constantino Miguel, Giovanni A Salum, Carolina Ziebold, Ary Gadelha

**Affiliations:** Department of Psychiatry, Federal University of São Paulo, São Paulo, Brazil; Department of Psychiatry, Interdisciplinary Laboratory in Clinical Neuroscience (LiNC), Department of Psychiatry, Federal University of São Paulo, São Paulo, Brazil; Department of Psychiatry, Federal University of São Paulo, São Paulo, Brazil; Department of Psychiatry, Interdisciplinary Laboratory in Clinical Neuroscience (LiNC), Department of Psychiatry, Federal University of São Paulo, São Paulo, Brazil; Department of Psychiatry, Federal University of São Paulo, São Paulo, Brazil; Department of Psychiatry, Interdisciplinary Laboratory in Clinical Neuroscience (LiNC), Department of Psychiatry, Federal University of São Paulo, São Paulo, Brazil; Department of Psychiatry, Schizophrenia Program (PROESQ), Federal University of São Paulo, Sao Paulo, Brazil; Department of Psychiatry, Interdisciplinary Laboratory in Clinical Neuroscience (LiNC), Department of Psychiatry, Federal University of São Paulo, São Paulo, Brazil; National Institute of Developmental Psychiatry for Children and Adolescents (INCT-CNPq), São Paulo, Brazil; Department of Psychiatry, Attention Deficit and Hyperactivity Disorder (ADHD) Outpatient and Developmental Psychiatry Programs, Federal University of Rio Grande do Sul, Porto Alegre, Brazil; Medical Council, UniEduK, São Paulo, Brazil; Department and Institute of Psychiatry (IPQ), University of São Paulo, São Paulo, Brazil; National Institute of Developmental Psychiatry for Children and Adolescents (INCT-CNPq), São Paulo, Brazil; Child Mind Institute, New York, USA; Department of Psychiatry, Federal University of São Paulo, São Paulo, Brazil; Department of Psychiatry, Federal University of São Paulo, São Paulo, Brazil; National Institute of Developmental Psychiatry for Children and Adolescents (INCT-CNPq), São Paulo, Brazil

**Keywords:** attenuated psychotic symptoms, early diagnosis, psychosis early markers, developmental psychopathology, cross-lagged panel models

## Abstract

**Background and Hypothesis:**

When occurring in adolescence, psychotic experiences (PE), subclinical psychotic symptoms, can be an early marker of mental illnesses. Studies with high-risk populations for psychosis show that anxiety symptoms often precede the onset of psychosis. Although anxiety symptoms are frequently experienced across the continuum of psychosis, no previous study has analyzed this association using a cross-lagged panel model (CLPM) longitudinally to identify if anxiety can be a predictor of PE over time or vice versa. The aim of the current study was to investigate whether one symptom domain predicts the other over time.

**Study Design:**

2194 children from the Brazilian High-Risk Cohort (BHRC) were evaluated at baseline (*T*_0_), and 76.5% completed a 3-year follow-up (*T*_1_) interview. Childhood anxiety symptoms and PE were assessed using a standardized self-report questionnaire at both time points. Cross-lagged panel models evaluated time-lagged associations between PE and anxiety longitudinally.

**Study Results:**

Higher levels of anxiety in childhood predicted an increase in PE levels in adolescence. The cross-lagged effect of anxiety scores at *T*_0_ on PE scores at *T*_1_ was significant (β = .03, SE = 0.01, *P* ≤ .001) and PE in childhood did not increase levels of anxiety in adolescence, when controlling for sociodemographic and clinical characteristics.

**Conclusions:**

Our findings reinforce that anxiety may represent an early marker of psychosis proneness, not a consequence of already presenting PE, which can help to develop better screening approaches. Therefore, future studies should focus on identifying biological or other clinical markers to increase prediction accuracy.

## Introduction

Psychotic experiences (PE) such as subclinical delusions and/or hallucinations are usually transient in childhood.^1^ However, cross-sectional studies have shown that during adolescence, up to 80% of subjects with PE also present a comorbid psychiatric disorder,^2–5^ especially anxiety symptoms.^6,7^ Longitudinal studies have expanded these findings, revealing that adolescents who experienced PE over 1–2-year follow-ups presented either (1) worsening of previous depression/anxiety symptoms or (2) its persistence even after PE remission.^8–11^ Furthermore, several prospective cohort studies indicate that juvenile presentation of PE is associated with anxiety disorder as well as psychotic disorders in adulthood.^12–14^ Understanding the course of anxiety and psychotic symptoms over development can be useful to improve screening assessments of initial mental illness manifestations, in a timely manner as to adopt preventive measures or early treatment.

Studies with high-risk populations for psychosis show that anxiety symptoms often precede the onset of psychosis,^15–19^ especially if they are persistent during childhood.^20–22^ Moreover, individuals who develop psychosis are more likely to experience anxiety, with prevalence rates for anxiety disorders in psychosis ranging from 42% to 74%.^23,24^ Although psychotic symptoms are often experienced with anxiety symptoms across the continuum of psychosis,^10,15–17,25^ it remains unclear whether one leads to the other throughout development.

Psychotic experiences and anxiety symptoms can be early markers of various psychiatric disorders,^17–19,26–28^ but they might also influence each other. In addition to sharing common pathways, anxiety symptoms are thought to play a central role in the formation of psychotic symptoms according to cognitive models of psychosis.^29^ Thus, despite the growing number of longitudinal studies that show an association between PE and anxiety symptoms, some methodological aspects limit our understanding of the association between anxiety and PE. For instance, lack of adequate methods to consider the autocorrelation of repeated measures and to control for potential confounders, such as low socioeconomic status, cognitive impairment, and family history of mental illness,^11,30,31^ make it difficult to disentangle the contributions of anxiety to PE, and vice versa.

Cross-lagged panel models (CLPMs) can be useful to investigate the longitudinal association between PE and anxiety since it can estimate to what extent the prior presence of one relates to the other adjusted for (1) autoregressive effects of the same measure over time (eg, childhood anxiety on adolescent anxiety), (2) correlation between both measures, anxiety and PE, at each time point, and (3) potential confounders.^32^ As such, this study investigated the relationship between anxiety symptoms and PE across childhood and adolescence employing CLPMs. We used data from the Brazilian High-Risk Cohort for Psychiatric Disorders (BHRC), a school-based cohort of young people that completed a follow-up assessment over 3 years.^33^ The specific aims of the current study were (1) to investigate if there is an association between PE and anxiety over time and (2) whether one symptom domain predicts the other over time.

A longitudinal study in Sweden^31^ has shown that PE predicts anxiety symptoms three years later in adolescents (mean age of 14 years old at baseline). Analyzing the same age range, a 5-year survey in Japan,^11^ using multilevel model analysis, revealed that anxiety worsened with PE incidence in adolescents and that PE incidence was associated with increased anxiety symptoms. Morales-Muñoz et al,^20^ conducted latent class growth analyses for anxiety using data from a cohort starting at 8 years old along three time points (8, 10, and 13 years old), where persistent high levels of anxiety across time points were significantly associated with PE at the age of 24 years.

The results of these studies show that anxiety and PE are associated over time. However, none of them used a CLPM to analyze whether, in a younger age group (6–12 years at baseline), one of the symptoms may precede the other. Accordingly, elucidating if there is an initial symptom, which appears earlier in development and predict the other in adolescence, is of great importance for early prevention and to better understand the transition from PE to mental health issues.

## Methods

### Study Design and Subjects

Our study used information from two time-point assessments (*T*_0_ in 2010/2011 and *T*_1_ in 2014/2015) of the BHRC.^33^ For a detailed description of study procedure, please see Salum *et al*.^33^ Briefly, on the registration day, 12 500 parents with children aged between 6 and 12 years, who were enrolled with 57 schools (22 in Porto Alegre and 35 in São Paulo), were asked to participate in a screening interview utilizing the Family History Screen (FHS).^34^ FHS is a structured interview conducted by lay interviewers in which parents provide information about the presence of lifetime DSM-IV major mental disorder in each of the biological first-degree relatives. A total of 8012 families (9937 eligible children and 45 394 family members) underwent FHS interviews. In 87% of cases, the biological mother was the primary informant. For each potential eligible child, a family load index was calculated, considering the percentage of family members screening positively for the evaluated disorders, adjusted for relatedness. Finally, the cohort was comprised of 2511 individuals, children and adolescents, of which 957 were randomly selected, and 1554 consisted of a selection of children identified as at high risk of mental disorders through the Family History Screen (FHS). Child assent and parental written informed consent were obtained from all research subjects.

We analyzed data from subjects who completed the assessment of PE at baseline and who had an estimated intelligence quotient (IQ) above 70. IQ was estimated using the vocabulary and block design subtests of the Weschler Intelligence Scale for Children, 3rd edition—WISC-III,^35^ according to the Tellegen and Briggs method^36^ and Brazilian standards.^37^ The sample of the present study comprises 2194 individuals at baseline (*T*_0_) and 1678 at the follow-up, *T*_1_.

### Assessments of PE

To assess PE, the Community Assessment of Psychic Experiences (CAPE),^38^ specifically formulated to evaluate the frequency and impact of psychotic subclinical symptoms, was used. The original scale consists of 42 self-report items, distributed across positive, negative, and depressive dimensions. Only the 20 items of positive symptom subscale (CAPE-pos) were applied to students in our cohort. CAPE-pos is reliable among young people (mean age ≤25 years)^39^ and demonstrated satisfactory factor validity as well as reliability coefficient to assess PE.^38,40^ Considering the initial age range of our sample (6–12 years-old), the questionnaire was applied by trained psychologists.

At both time points, the frequency of PE was quantified using a four-point Likert scale (0 = never, 1 = sometimes, 2 = often, and 3 = nearly always). In the current study, the sum of the frequencies of all 20 CAPE-pos items was used as a continuous variable, ranging from 0 to 60.

### Assessing Anxiety Symptoms

To assess anxiety symptoms, we used the Screen for Child Anxiety Related Emotional Disorders (SCARED), a self-report questionnaire used to measure anxiety in children and adolescents.^41–47^ The scale consists of 41 items divided into five categories of symptoms: panic/somatic (13 items); generalized anxiety (9 items); separation anxiety (8 items); social phobia (7 items); and school avoidance (4 items). For each item, participants endorsed a 3-point Likert scale (0 = not true or hardly ever true; 1 = sometimes true; 2 = true or often true), which describes how they have been feeling in the past 3 months. Total scores, therefore, ranged from 0 to 82, with higher scores reflecting higher levels of anxiety. The SCARED has been validated to Brazilian Portuguese and showed good reliability as measured by internal consistency and test–retest reliability.^48^ Birmaher *et al*,^42^ identified total scores at 25 or above as the cut-off score warranting further evaluation.

### Assessment of Covariates

#### Demographic Characteristics

Age, sex, city, socioeconomic status, and skin color data were collected. Socioeconomic status (SES) was obtained from a questionnaire on household assets and educational background of the household head. According to Brazil Criterion for Economic Classification,^49^ (ABEP) scores range from 0 to 46, where greater scores indicate higher socioeconomic class. Self-reported skin color was divided into two groups: White and Non-White. Non-White included people with black skin, mixed-race, Asian, and Indigenous.

#### General Psychopathology

Child Behavior Checklist (CBCL) is an inventory answered by parents, and it allows dimensional measurement of behavioral problems of internalization (withdrawal, anxiety, depression, and somatic complaints) and externalization (aggressiveness and challenging behavior) symptoms in children and adolescents. To assess general psychiatric symptoms, CBCL total score was used, consisting of a sum of 118 items classified as “not true” (0), “partially or sometimes true” (1), or “very true or often true” (2).^50^

#### Parental Diagnosis

Main caregiver (biological mother in 95% of cases and biological father in 5%) was assessed for psychopathology at baseline through the Mini International Neuropsychiatric Interview (MINI),^51^ which investigated anxiety (panic, agoraphobia, social, or generalized anxiety disorder), mood (recurrent depression, bipolar, and unipolar depression), substance use (alcohol dependence or abuse, drug dependence, or abuse), psychotic disorders and attention deficit hyperactivity disorder (ADHD).

### Statistical Analysis

We first present descriptive statistics of the research variables at each assessment and the correlation between all variables included in the study. Logistic regression models were employed to investigate predictors of attrition at *T*_1_. Then, longitudinal association between PE and anxiety was assessed using CLPMs in Mplus version 8.6.^52^ CLPMs are used to investigate the interdependence of variables over time. Time-lagged associations between PE (CAPE total score) and anxiety (SCARED scores) was longitudinally examined at *T*_0_ and *T*_1_. CLPMs allow to estimate the cross-lagged effect that anxiety and PE have over time or, in other words, to what extent the prior scores of one variable relate to subsequent scores of the other variable adjusted by (1) autoregressive effects of the same measure over time (eg, anxiety at *T*_0_ on anxiety at *T*_1_), (2) correlation between both measures, anxiety, and PE, at each time point, and (3) potential confounders.^32^ We used the Maximum Likelihood with robust standard errors (MLR) estimator that provides a robust estimator when continuous variables are not normally distributed and allows to handle missing data using all available information even if some data points may be absent, yielding robust standard error estimation.^52^ We first estimated a baseline model without adjustments. We then estimated an adjusted model including time invariant (gender, site, skin color, parental diagnosis at baseline) and time variant covariates (CBCL scores, ABEP score, and age at each time point). We compared the performance of both models using the Information Criteria Akaike (AIC), Bayesian Information Criteria (BIC), and Sample-Size Adjusted BIC (saBIC), where the model with lower indexes indicates a better goodness of fit. Additionally, we adopted a significant level of 5%. Standardized estimates were presented for the CLPMs. Significant standardized estimates between <0.20, 0.20–0.49 and >0.50 were interpreted as representing small, medium, and large effect sizes, respectively.^53^

For a sensitivity analysis, we tested whether general psychopathology better explains the prediction of PE longitudinally than anxiety alone, thus using general psychopathology (as measured by CBCL) as a predictor, instead of anxiety (measured by SCARED).

## Results

### Sample Characteristics

Table 1 presents sociodemographic and clinical characteristics in both assessments. Details on current parental diagnosis at baseline are shown in Supplementary table 2. The most prevalent lifetime diagnosis among parents was anxiety disorder (*n* = 521, 23.8%) followed by mood disorder (*n* = 435, 19.8%). Baseline characteristics associated with attrition at *T*_1_ were female gender (OR = 1.27, 95% CI = 1.04–1.54, *P* = .019), higher age (OR = 1.08, 95% CI = 1.02–1.13, *P* = .005), and lower SES scores (OR = 1.03, 1.01–1.05, *P* = .016). Clinical characteristics (CBCL, PE, anxiety scores, parental diagnosis, and family risk of psychiatric disorders), skin color, and site were not significantly associated with attrition. Supplementary table 3 presents the correlation matrix between all study variables.

**Table 1.** Sample Characteristics

	Mean (SD) or *n* (%)	
	*T*_0_ (*n* = 2194)	*T*_1_ (*n* = 1678)
Demographics*		
Age (years)	10.2 (1.91)	13.44 (1.91)
ABEP score	18.3 (4.51)	18.44 (4.29)
Gender		
Male	1196 (54.5)	938 (55.9)
Female	998 (45.5)	740 (44.1)
Selection		
Familiar high-risk	1346 (61.3)	1026 (61.1)
Random	848 (38.7)	652 (38.9)
Site		
São Paulo	1090 (49.7)	827 (49.3)
Porto Alegre	1104 (50.3)	851 (50.7)
Skin color^†^		
White	1331 (60.8)	1023 (61.1)
Non-White	857 (39.2)	650 (38.9)
Parental diagnosis		
Yes	654 (29.8)	517 (30.8)
No	1540 (70.2)	1161 (69.2)
General psychopathology (CBCL)	26.3 (24.54)	26.47 (22.92)
Psychotic experience (CAPE)	4.81 (5.45)	3.15 (4.20)
Anxiety symptoms (SCARED)	24.48 (14.2)	20.5 (11.61)

*Note*: ABEP, The Brazil Criterion for Economic Classification; CAPE, Community Assessment of Psychic Experiences; CBCL, Child Behavior Checklist; SCARED, Screen for Child Anxiety Related Emotional Disorders.

*Only data from subjects with complete assessment of psychotic experiences (CAPE-pos) at *T*_0_ were used in this study.

^†^Skin color | *T*_0_ (*n* = 2188) | *T*_1_ (*n* = 1673).

### Psychotic Experience

In our sample, at the baseline (*T*_0_), 1634 (74.50%) individuals reported at least one PE, with median of 3 endorsements; and 41.5% endorsed at least one experience as “‘often’” or “‘almost always’.” At the follow-up (*T*_1_), 1197 (71.33%) reported at least one PE, with median of 2 endorsements; and 30.20% endorsed at least one experience as “often” or “almost always.” The CAPE total score ranged from 0 to 35 (mean = 4.81, SD = 5.45) at *T*_0_ and from 0 to 37 (mean = 3.15, SD = 4.20) at *T*_1_ (table 1).

### Anxiety Symptoms

The total score for the entire sample ranged from 0 to 82 at *T*_0_ (mean = 24.48, SD = 14.20) and from 0 to 81 at *T*_1_ (mean = 20.50, SD = 11.61) (table 1). Approximately 43.5% (*n* = 943) of the sample at *T*_0_ and 30% (*n* = 510) at *T*_1_ had total scores at 25 or above.

### Cross-lagged Panel Models

**Fig. 1.**
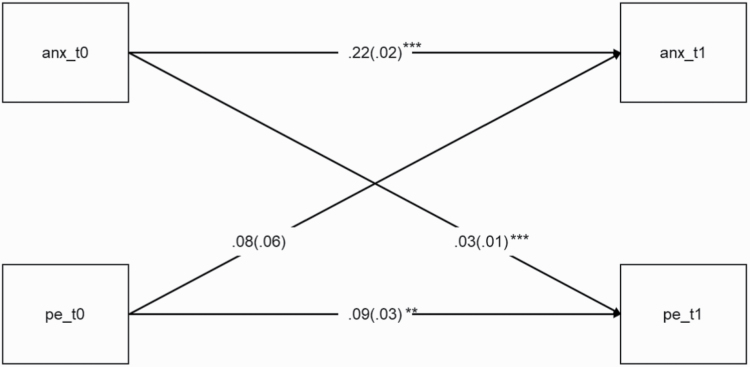
Cross-lagged panel model: interrelationship between anxiety and psychotic experiences over childhood and adolescence. Anxiety symptoms: Anxiety symptoms: anx_t0, anx_t1 = anxiety total score at Time 0 and 1, respectively. Psychotic Experiences: pe_t0, pe_t1 = PE total score at Time 0 and 1, respectively. Standardized estimates are shown, error terms were omitted for visual clarity. ***P value < .001. **P value < .05. The model was adjusted for age, gender, parental diagnosis, site, CBCL scores, ABEP score, and skin color as showed in table 2. Covariance pe_t0 and anx_t0 = 21.10, *P* < .001, pe_t1 and anx_t1 = 17.10, *P* < .001. Model Fit information: Number of Free Parameters = 28; Loglikelihood H0 Value = −26467.58; Information Criteria Akaike (AIC) = 52991.16; Bayesian (BIC) = 53150.50, Sample-Size Adjusted BIC = 53282.43. *n* = 2188. Estimator: MLR.

As shown in Supplementary figure 1, the non-adjusted model showed that the cross-lagged effect of anxiety at *T*_0_ on PE at *T*_1_ was small, but significant (β = .04, SE = 0.01, *P* < .001). The covariance between both measures at each time point was significant, as well as the autoregressive effect of each variable on its own development over time (figure 1). The adjusted model is shown in figure 1 and a complete description of results can be found in table 2. This model presented better goodness of fit (AIC = 52991.156, BIC = 53150.497, saBIC = 53061.537) compared to the model without adjustments (AIC = 53202.718, BIC = 53282.426, and sample-size adjusted BIC = 53237.946).

**Table 2.** Standardized Estimates of the Adjusted Cross-lagged Panel Model (*n* = 2188)

	Standardized Estimate	Standard Error	*P* values
Outcome: SCARED *T*_1_			
CAPE *T*_0_	0.076	0.057	.185
SCARED *T*_0_	0.224	0.022	<.001
Age *T*_0_	−0.106	0.146	.468
ABEP score *T*_0_	−0.115	0.056	.042
Gender	2.691	0.559	<.001
Site	0.479	0.574	.404
Skin color	−0.168	0.561	.764
Parental diagnosis	1.934	0.655	.003
CBCL *T*_0_	0.019	0.012	.125
CAPE *T*_0_	0.076	0.057	.185
SCARED *T*_0_	0.224	0.022	<.001
Outcome: CAPE *T*_1_			
CAPE *T*_0_	0.090	0.028	.001
SCARED *T*_0_	0.034	0.008	<.001
Age *T*_0_	−0.014	0.055	.793
ABEP score *T*_0_	−0.030	0.021	.160
Gender	0.743	0.206	<.001
Site	1.130	0.206	<.001
Skin color	−0.004	0.216	.986
Parental diagnosis	−0.078	0.234	.740
CBCL *T*_0_	0.017	0.005	.001

*Note*: CAPE, Community Assessment of Psychic Experiences; SCARED, Screen for Child Anxiety Related Emotional Disorders; ABEP, Brazil Criterion for Economic Classification; CBCL, Child Behavior Checklist.

In this model, the cross-lagged effect of anxiety scores at *T*_0_ on PE scores at *T*_1_ remained significant (β = .03, SE = 0.01, *P* ≤ .001), which means that each standard deviation increase in anxiety scores during childhood (*T*_0_) was associated with an increase by 0.03 standard deviations in PE during early adolescence (*T*_1_), even after controlling for sociodemographic and clinical characteristics. The cross-lagged effect of PE scores at *T*_0_ on anxiety scores at *T*_1_ was not significant.

For the sensitivity analysis, the result shows that general psychopathology at *T*_0_ also predicts PE at *T*_1_; however, in lower magnitude, half as much as anxiety. As shown in Supplementary table 1, the cross-lagged effect of general psychopathology at *T*_0_ on PE at *T*_1_ was significant (β = .017, SE = 0.005, *P* = .001).

## Discussion

Our study used the CLPM, a relevant analysis for literature, to investigate the direction of the association over time between anxiety and PE. We found a small but statistically significant effect of childhood anxiety symptoms on PE prediction in adolescence and that, surprisingly, PE in childhood did not predict anxiety in adolescence. Furthermore, the present study found that PE and anxiety are associated at each time point, and both represent the best predictor of their subsequent levels over time.

For a sensitivity analysis, we tested whether general psychopathology better explains the prediction of PE longitudinally than anxiety alone, thus using general psychopathology (as measured by the CBCL) as a predictor in the cross-lagged model, instead of anxiety (measured by the SCARED). We aimed to check the specificity of the association and if general psychopathology, also previously associated with PE,^54^ would account for the result we found with the anxiety measurement. The result showed that general psychopathology at *T*_0_ also predicted PE at *T*_1_, but its effect was half of that found for anxiety symptoms, suggesting a stronger sign of anxiety specifically. We cannot rule out that other specific psychopathological domains also predict PE, and testing each possibility is beyond our current scope. Interestingly, anxiety is a general response to distress and even other specific domains of psychopathology-linked PE may be accompanied by higher levels of anxiety. In addition, the result highlights the relevance of anxiety symptoms as an early marker of developing PE in adolescence.

We found that anxiety at *T*_0_ predicts PE at *T*_1_; however, it should be emphasized that the strongest predictor of PE at follow-up was PE at baseline; it also applies to anxiety, the best predictor of anxiety at *T*_1_ was anxiety at *T*_0_, which is consistent with previous study showing that the best predictor of a specific psychopathology on follow-up is the same psychopathology at baseline.^31^

Our finding that anxiety predicts psychosis is in line with previous literature where anxiety has been over-reported by children who later develop psychosis.^21,22^ This prediction can be understood from several points of view. The neurophysiology of anxiety and psychosis symptoms share common pathways. Those experiencing PE likely reflect a heightened tendency to avoid aversive or threatening stimuli comparable to what occurs in anxiety disorders.^55^ Moreover, a cross-sectional study has shown that schizophrenia patients are more propense to experience anxiety. This propensity is considered a vulnerability that may play a role in developing psychotic disorders.^56^

Giocondo *et al*^57^ study showed that dimensional measurement of PE, but not categorical, was associated with anxiety disorders in a 3-year follow-up of the same cohort. The method used for the analysis—independent linear/logistic regression models—do not control for auto-regressive effects in longitudinal designs, being unable to clarify the directionality of the results. In addition, the use of a categorical approach to anxiety reduces the power to identify milder relationship between anxiety and PE, as measured at the symptom level. In this sense, the current study aimed specifically to clarify the direction of the relationship between PE and anxiety symptoms over time, providing more insights about the pressing issue related to prediction, a crucial step to preventive protocols.

Our results contrast with those from Isaksson *et al*^31^ study, which shows that PE predicts anxiety and depression symptoms three years later, even adjusting for psychiatric symptoms at baseline. However, the age group studied was 14 to 17 years old (older than our cohort). The self-report questionnaire of children as young as 8 years old might lead to an overestimation of PE report compared to an interview-based survey and the sensitivity of the frequency self-report of PE is low at younger ages.^58^ Even so, we were able to replicate previously stablished associations between PE and anxiety on both time points as well as the autoregressive effect of each variable on its own development over time.

The findings of this study should be interpreted in the light of some limitations. First of all, the cohort follow-up for 3 years limits our ability to comment whether PE in adolescence predicts psychiatric illness in adulthood. Our latest assessment does not cover the entire age range of psychiatric disorder onset in adulthood. Secondly, besides CLPM can be a robust data analysis method, the presence of unmeasured confounding factors, such as trauma and substance use (cannabis), is always possible and could undermine the estimates presented in this study. Thirdly, given that the PE score is an asymmetric measure, skewed towards lower scores, for statistical analysis we used the Maximum Likelihood with robust standard errors (MLR) estimator that provides a reliable estimate in cases where continuous variables deviate from normal distribution. Furthermore, the occurrence of PE in our study aligns with prior research indicating a high prevalence of low-frequency PE, with a notable decrease in prevalence rates as the frequency increases.^27,59–62^ Finally, the effect of anxiety at *T*_0_ on PE at *T*_1_ is significant, but of small effect-size, which is not surprising, given that, despite of the high family risk criteria for the cohort, 74% of the sample did not have any psychiatric disorder,^33^ depicting lower levels of psychopathology among these children. Moreover, many other variables may be involved in the PE formation process, such as childhood trauma, substance use, among others.^10,63–65^

Strengths of our study include the contribution to literature by exploring the association of anxiety and PE using CLPMs, which is the ideal method to analyze longitudinally both variables and estimate the directional effects that one variable has on another. In addition, CLPM uses a robust estimator that allows the use of maximum available information, so there was minimal data loss. Considering a longitudinal study, we captured information from 2194 individuals without matching controls and 2188 individuals with controls due to the limitation of skin color data. To our knowledge, no previous study has shown this association using the same method. Moreover, our study analyzed data from a longitudinal high-risk cohort, carefully interviewed by trained psychologists. We used standardized scales to assess anxiety symptoms and PE, addressing concerns regarding the reliability of children’s self-report to PE.

Considering that all of the studies that have investigated the link between anxiety and PE have adopted cross-sectional designs or short-term longitudinal follow-up, future studies would benefit from using long-term longitudinal designs to examine, through CLPMs, the temporal nature of the relationships among childhood anxiety symptoms, PE during adolescence, and development of psychiatric illness in adulthood.

Anxiety has been identified as part of the initial prodrome in psychosis^66^ and a strong predictor of both development and persistence of paranoid thinking.^18^ Beyond that, prospective studies with high-risk populations during teenage years have shown that anxiety predicts, with some level of accuracy, psychotic disorders before their onsets.^15–19,21,22^ The present study replicates this finding longitudinally, with children aged from 6 to 12 years old whose anxiety increases the development of PE 3 years later and draws attention to early recognition and interventions.

## Supplementary Material

sgae003_suppl_Supplementary_Tables_2

sgae003_suppl_Supplementary_Tables_3

sgae003_suppl_Supplementary_Figures_1

sgae003_suppl_Supplementary_Tables_1
