# Supplementary material for: Childhood Anxiety Symptoms as a Predictor of Psychotic Experiences in Adolescence in a High-Risk Cohort for Psychiatric Disorders
Source: Schizophr Bull Open. 2024 Apr 15;5(1):sgae003. doi: 10.1093/schizbullopen/sgae003 (PMC11207689; doi:10.1093/schizbullopen/sgae003)
Supplement: sgae003_suppl_Supplementary_Tables_2 [file sgae003_suppl_Supplementary_Tables_2.docx]

**Supplementary Table 2 |** Spearman's Rank correlation coefficients and p values

|  | **Gender** | **Skin color**  **T_0_** | **Parental diagnosis T_0_** | **Age**  **T_0_** | **ABEP score**  **T_0_** | **Center** | **CBCL T_0_** | **SCARED T_0_** | **SCARED T_1_** | **CAPE**  **T_0_** | **CAPE**  **T_1_** |
| --- | --- | --- | --- | --- | --- | --- | --- | --- | --- | --- | --- |
| **Gender** | 1.00 |  |  |  |  |  |  |  |  |  |  |
| **Skin color T_0_** | 0.01 | 1.00 |  |  |  |  |  |  |  |  |  |
| p-value | 0.66 |  |  |  |  |  |  |  |  |  |  |
| **Parental diagnosis T_0_** | 0.04 | 0.02 | 1.00 |  |  |  |  |  |  |  |  |
| p-value | 0.10 | 0.38 |  |  |  |  |  |  |  |  |  |
| **Age T_0_** | 0.03 | -0.02 | 0.05 | 1.00 |  |  |  |  |  |  |  |
| p-value | 0.30 | 0.32 | 0.03 |  |  |  |  |  |  |  |  |
| **ABEP score T_0_** | -0.05 | -0.11 | -0.05 | 0.03 | 1.00 |  |  |  |  |  |  |
| p-value | 0.05 | <0.001 | 0.05 | 0.17 |  |  |  |  |  |  |  |
| **Center** | -0.05 | 0.12 | -0.21 | -0.12 | 0.07 | 1.00 |  |  |  |  |  |
| p-value | 0.03 | <0.001 | <0.001 | <0.001 | <0.001 |  |  |  |  |  |  |
| **CBCL T_0_** | -0.06 | 0.02 | 0.37 | 0.04 | -0.07 | -0.20 | 1.00 |  |  |  |  |
| p-value | 0.02 | 0.45 | <0.001 | 0.08 | <0.001 | <0.001 |  |  |  |  |  |
| **SCARED T_0_** | 0.16 | 0.03 | 0.02 | 0.00 | -0.05 | 0.05 | 0.07 | 1.00 |  |  |  |
| p-value | <0.001 | 0.28 | 0.37 | 0.93 | 0.04 | 0.03 | 0.01 |  |  |  |  |
| **SCARED T_1_** | 0.15 | 0.01 | 0.10 | -0.01 | -0.06 | 0.01 | 0.08 | 0.31 | 1.00 |  |  |
| p-value | <0.001 | 0.72 | <0.001 | 0.66 | 0.02 | 0.55 | <0.001 | <0.001 |  |  |  |
| **CAPE T_0_** | 0.09 | 0.05 | -0.02 | -0.06 | -0.08 | 0.19 | -0.02 | 0.25 | 0.11 | 1.00 |  |
| p-value | <0.001 | 0.03 | 0.46 | 0.03 | <0.001 | <0.001 | 0.34 | <0.001 | <0.001 |  |  |
| **CAPE T_1_** | 0.10 | 0.03 | -0.01 | 0.04 | -0.01 | 0.15 | 0.04 | 0.19 | 0.37 | 0.15 | 1.00 |
| p-value | <0.001 | 0.20 | 0.80 | 0.07 | 0.80 | <0.001 | 0.11 | <0.001 | <0.001 | <0.001 |  |

Note: ABEP, The Economic Classification Criterion Brazil; CAPE, Community Assessment of Psychic Experiences (Psychotic Experience); CBCL, Child Behavior Checklist (General Psychopathology); SCARED, Screen for Child Anxiety Related Emotional Disorders (Anxiety symptoms).
