# Supplementary material for: Childhood Anxiety Symptoms as a Predictor of Psychotic Experiences in Adolescence in a High-Risk Cohort for Psychiatric Disorders
Source: Schizophr Bull Open. 2024 Apr 15;5(1):sgae003. doi: 10.1093/schizbullopen/sgae003 (PMC11207689; doi:10.1093/schizbullopen/sgae003)
Supplement: sgae003_suppl_Supplementary_Tables_3 [file sgae003_suppl_Supplementary_Tables_3.docx]

**Supplementary Table 3** | Cross-lagged Panel Model: Interrelationship between general psychopathology and psychotic experiences over childhood and adolescence with adjustments

|  | **Standardized Estimate** | **Standard Error** | **P-Value** |
| --- | --- | --- | --- |
| **Outcome: CBCL T_1_** |  |  |  |
| CBCL T_0_ | **0.368** | **0.026** | **0.000** |
| CAPE T_0_ | -0.030 | 0.095 | 0.756 |
|  |  |  |  |
| **Outcome: CAPE T_1_** |  |  |  |
| CBCL T_0_ | **0.017** | **0.005** | **0.001** |
| CAPE T_0_ | **0.090** | **0.028** | **0.001** |
|  |  |  |  |
|  |  |  |  |

Note: the model was adjusted by anxiety, age, gender, parental mental health diagnosis, site, socioeconomic status and skin color. CAPE, Community Assessment of Psychic Experiences; CBCL, Child Behavior Checklist.
