# Supplementary material for: Childhood Anxiety Symptoms as a Predictor of Psychotic Experiences in Adolescence in a High-Risk Cohort for Psychiatric Disorders
Source: Schizophr Bull Open. 2024 Apr 15;5(1):sgae003. doi: 10.1093/schizbullopen/sgae003 (PMC11207689; doi:10.1093/schizbullopen/sgae003)
Supplement: sgae003_suppl_Supplementary_Figures_1 [file sgae003_suppl_Supplementary_Figures_1.docx]

**Supplementary Figure 1** | Cross-lagged Panel Model: Interrelationship between anxiety symptoms and psychotic experiences over childhood and adolescence without adjustments


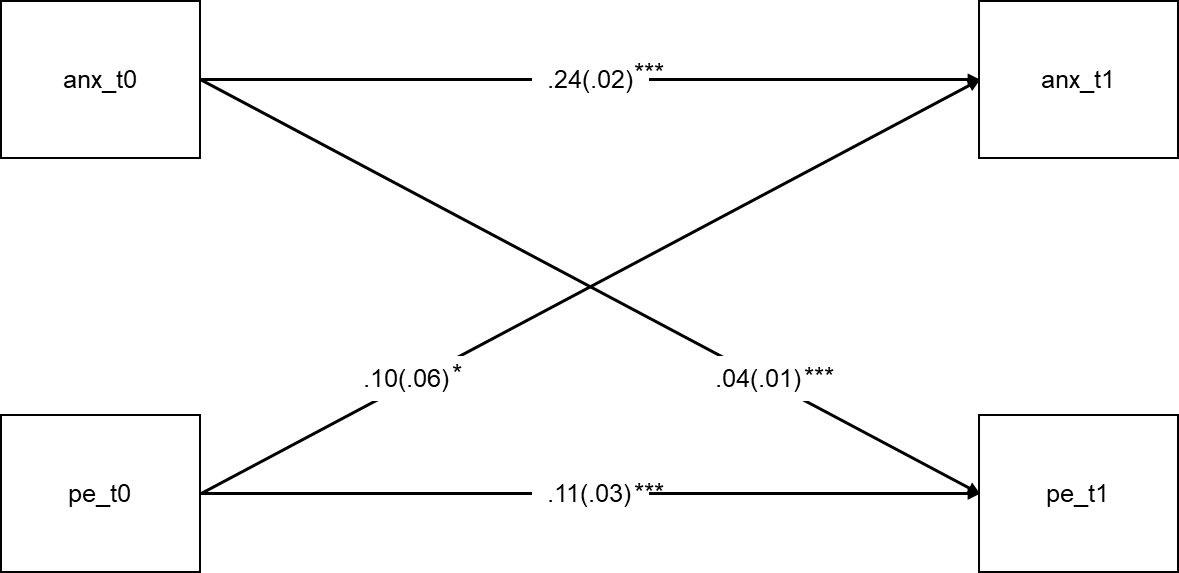


Notes: Anxiety symptoms: anx_t0, anx_t1= anxiety total score at Time 0 and 1, respectively. Psychotic Experiencies: pe_t0, pe_t1= PE total score at Time 0 and 1, respectively. Standardized estimates and standard errors are shown, error terms were omitted for visual clarity. ***: p value<0.001. *p value<0.10. N=2194. Covariance pe_t0 and anx_t0=21.09, p<0.001, pe_t1 and anx_t1=17.80, p<0.001. Model Fit information: Number of Free Parameters=14; Loglikelihood H0 Value= -26587.36; Akaike Information Criteria (AIC)= 53202.72; Bayesian (BIC) = 53282.43, Sample-Size Adjusted BIC= 53237.95.
