# Supplementary material for: Childhood Anxiety Symptoms as a Predictor of Psychotic Experiences in Adolescence in a High-Risk Cohort for Psychiatric Disorders
Source: Schizophr Bull Open. 2024 Apr 15;5(1):sgae003. doi: 10.1093/schizbullopen/sgae003 (PMC11207689; doi:10.1093/schizbullopen/sgae003)
Supplement: sgae003_suppl_Supplementary_Tables_1 [file sgae003_suppl_Supplementary_Tables_1.docx]

**Supplementary Table 1 |** Parental diagnosis*

| **Diagnosis** | **N (2,194)** | **%** |
| --- | --- | --- |
|  |  |  |
| **Anxiety disorder** |  |  |
| Generalized anxiety disorder | 366 | 16.68 |
| Agoraphobia | 255 | 11.62 |
| Panic | 160 | 7.29 |
| Social Anxiety | 134 | 6.11 |
|  |  |  |
| **Mood disorder** |  |  |
| Unipolar depression | 317 | 14.45 |
| Recurrent depression | 222 | 10.12 |
| Bipolar depression | 68 | 3.10 |
|  |  |  |
| **Substance use** |  |  |
| Alcohol Dependence | 11 | 0.50 |
| Alcohol Abuse | 6 | 0.27 |
| Drug Dependence | 9 | 0.41 |
| Drug Abuse | 9 | 0.41 |
|  |  |  |
| **Psychotic Syndrome** | 109 | 4.97 |
|  |  |  |
| **ADHD** | 3 | 0.14 |

Note: ADHD, Attention Deficit and Hyperactivity Disorder.

* The main caregiver psychopathology, 95% of biological mother and 5% biological father.
